# Prevalence of self-reported shoulder pain and shoulder function among uninjured CrossFit athletes in Pretoria, South Africa

**DOI:** 10.17159/2078-516X/2026/v38i1a25440

**Published:** 2026-06-15

**Authors:** M Dawood, E Meyer, TP Moatshe

**Affiliations:** 1 Department of Physiotherapy, Sefako Makgatho Health Sciences University, Pretoria, South Africa; 2 Department of Physiotherapy, Faculty of Health Sciences, University of Pretoria, Pretoria, South Africa

**Keywords:** overhead athletes, resistance training, musculoskeletal injury, shoulder function, exercise-related pain

## Abstract

**Background:**

CrossFit is a rapidly growing sport characterised by high-intensity functional movements that place substantial demands on the shoulder complex. Although shoulder injuries are frequently reported, the prevalence of self-reported shoulder pain and shoulder function among uninjured CrossFit athletes remains unclear.

**Objectives:**

To determine the prevalence of self-reported shoulder pain and to describe shoulder function among uninjured CrossFit athletes in Pretoria, Gauteng.

**Methods:**

A cross-sectional study was conducted among CrossFit athletes recruited from gyms in Pretoria. Participants completed a sociodemographic and training questionnaire. Pain presence and severity were assessed using the Numeric Pain Rating Scale (NPRS), based on current shoulder pain intensity. Shoulder function was assessed using the Penn Shoulder Score (PSS). Descriptive statistics (means, standard deviations, frequencies, and percentages) were used to summarise the data. Chi-square tests were used to explore associations between shoulder pain and selected demographic and training-related variables.

**Results:**

Sixty-eight participants provided valid responses for pain analysis, while all participants completed the PSS. The prevalence of self-reported shoulder pain was 76% (95% CI: 64%–85%), with a mean NPRS score of 2.97 ± 2.75/10, indicating predominantly low-intensity pain. Shoulder function was generally preserved, with a mean total PSS of 87.8 ± 12.2 (out of 100). Shoulder pain was significantly associated with previous shoulder injury (p < 0.05). No significant associations were found between demographic or training-related variables and shoulder function.

**Conclusion:**

Self-reported shoulder pain is highly prevalent among uninjured CrossFit athletes despite largely preserved shoulder function. These findings suggest that athletes may continue training despite experiencing pain, highlighting the importance of routine screening and early intervention strategies.

CrossFit has emerged as a high-intensity training modality combining Olympic weightlifting, gymnastics, and metabolic conditioning. While its popularity continues to grow in South Africa, the high training volumes and frequent overhead movements place considerable mechanical demands on the shoulder complex. The shoulder joint is especially vulnerable due to its large range of motion and reliance on dynamic stabilisation. Consequently, shoulder pain is highly prevalent in overhead-dominant sports. In swimmers, prevalence estimates vary widely, ranging from approximately 20% to over 70%, with some reports suggesting values as high as 91%.^[1–3]^ Similar patterns have been observed in strength- and overhead-based sports such as Olympic weightlifting, gymnastics, and throwing sports, which share key biomechanical demands with CrossFit.^[4,7,10]^ These findings highlight the susceptibility of the shoulder to both repetitive loading and high-intensity training and provide an important reference point for investigating CrossFit populations.

In both recreational and competitive CrossFit cohorts from Europe and North America, the shoulder is consistently identified as one of the most affected anatomical regions. Injury rates of approximately 20% to 36% have been reported, with the shoulder frequently cited as a primary site of complaint.^[6,10,14]^ Importantly, many athletes continue training despite experiencing pain, suggesting that injury-based prevalence estimates may underestimate the true burden of shoulder-related symptoms in this population. Evidence from other sports further supports this distinction. Surveillance studies using the Oslo Sports Trauma Research Centre (OSTRC) methodology have reported a shoulder overuse prevalence of approximately 22% among elite handball players, with athletes continuing to train and compete despite ongoing symptoms.^[13]^ These findings emphasise the importance of epidemiological approaches that capture not only time-loss injuries but also ongoing pain and functional status.

Despite the growing body of international literature, there remains limited data on shoulder pain prevalence and shoulder function among CrossFit athletes in the South African context. Existing local studies have primarily focused on injury incidence rather than persistent symptoms or functional outcomes, leaving a gap in understanding the true burden of shoulder-related complaints in this population. Therefore, the aim of this study was to determine the prevalence of shoulder pain and to describe shoulder function among uninjured CrossFit athletes in Pretoria, Gauteng.

## Methods

### Study design

This study employed a cross-sectional descriptive design to determine the prevalence of shoulder pain and to describe shoulder function among uninjured CrossFit athletes in Pretoria, Gauteng.

For the purposes of this study, “uninjured” was defined as the absence of a current medical diagnosis or a time-loss injury (i.e., injury resulting in inability to participate in training) within the past three months. This definition aligns with established distinctions between time-loss and non–time-loss injuries, in which athletes may continue participating despite pain or symptoms.^[15]^

### Participants

A convenience sample of 70 CrossFit athletes was recruited from established CrossFit gyms in Pretoria. Permission was obtained from gym owners to recruit participants, after which athletes were invited to participate voluntarily.

Athletes were included if they were 18 years or older, had been actively training in CrossFit for at least six months, and reported no current medical diagnosis or acute musculoskeletal injury preventing participation in training. Athletes with recent (<3 months) shoulder surgery or those undergoing rehabilitation for an acute shoulder injury were excluded.

### Procedures

Participants provided written informed consent prior to data collection. Sociodemographic and training-related data were collected using a structured questionnaire, including age, sex, body mass index, CrossFit training frequency, years of experience, and weekly training hours.

### Shoulder-related outcomes assessed

#### Shoulder pain

Pain presence and severity were assessed using the Numeric Pain Rating Scale (NPRS), a validated 11-point scale ranging from 0 (no pain) to 10 (worst imaginable pain). Participants were asked to report their current shoulder pain intensity. Pain severity was categorised as low (1–3), moderate (4–6), and high (7–10).^[5]^

#### Shoulder function

Shoulder function was assessed using the Penn Shoulder Score (PSS), a validated patient-reported outcome measure used to assess shoulder pain, satisfaction, and functional ability. The PSS comprises three domains: pain (3 items assessing pain at rest, during normal activities, and during strenuous activities), satisfaction (1 item assessing overall satisfaction with shoulder function), and function (20 items assessing difficulty performing activities of daily living and upper-limb tasks). Each item is scored on a numerical scale, with domain scores summed to a total score ranging from 0 to 100. Higher scores indicate less pain, greater satisfaction, better functional ability, and better overall shoulder health.^[6]^

### Data management and statistical analysis

Data were captured in Microsoft Excel and analysed using SPSS version 29 (IBM Corp., Armonk, NY, USA).

Descriptive statistics were used to summarise the data. Continuous variables were reported as means and standard deviations, while categorical variables were presented as frequencies and percentages. These included participant demographics, training characteristics, shoulder pain prevalence, and shoulder function scores.

Associations between shoulder pain and selected demographic and training-related variables were examined using chi-square tests, with significance set at p < 0.05. Penn Shoulder Score outcomes were analysed descriptively as continuous variables. Prevalence estimates were calculated using valid responses for each outcome variable. Incomplete questionnaires were excluded from analyses where relevant data were missing.

### Ethical considerations

The study received ethical clearance from the Sefako Makgatho Health Sciences University Research Ethics Committee (Reference: SMUREC/H/384/2023). All participants provided written informed consent prior to participation. Data were collected anonymously and handled confidentially in accordance with the principles of the Declaration of Helsinki.

## Results

### Participants

A total of 70 CrossFit athletes were recruited. Two participants provided incomplete responses for shoulder pain, resulting in 68 valid responses for pain prevalence analyses. All participants completed the Penn Shoulder Score.

The mean age of participants was 32.9 ± 9.7 years, with 53% males (n = 36) and 47% females (n = 32). Participants reported a mean CrossFit training experience of 4.6 ± 3.1 years and trained an average of 5.0 ± 2.6 hours per week (Table 1).

### Prevalence of shoulder pain

Of the 68 participants with valid pain data, 52 athletes (76%) reported experiencing shoulder pain during CrossFit training, while 16 athletes (24%) reported no shoulder pain (Table 2).

### Pain intensity

Among participants reporting shoulder pain (n = 52), pain severity was predominantly low. The intensity of pain was measured using a numerical scale of 1–10. Low pain intensity being categorized by values (1–3), moderate pain (4–6) and high pain (7–10). Low-intensity pain (1–3/10) was reported by 28 athletes (41%), moderate pain (4–6/10) by 14 athletes (21%), and high pain (7–10/10) by 10 athletes (15%).

The mean NPRS score among participants reporting pain was 2.97 ± 2.75/10, indicating overall low pain intensity despite the high prevalence of reported shoulder pain (Table 2).

### Shoulder function (Penn Shoulder Score)

Shoulder function, assessed using the Penn Shoulder Score, was generally well preserved among participants (n = 70). The mean total PSS was 87.8 ± 12.2 (out of 100), indicating high self-reported shoulder function despite the high prevalence of shoulder pain.

Mean sub-scores were 26.0 ± 4.7 for the pain domain (0–30), 7.2 ± 2.7 for satisfaction (0–10), and 54.7 ± 6.9 for function (0– 60) (Table 3).

### Associations with training characteristics

Chi-square analyses demonstrated a significant association between shoulder pain and a history of shoulder injury (p < 0.05). No statistically significant associations were identified between shoulder pain and other demographic or training-related variables (p > 0.05).

No statistically significant associations were observed between Penn Shoulder Score outcomes and demographic or training-related variables (p > 0.05).

## Discussion

The present study found a high prevalence of shoulder pain (76%) among uninjured CrossFit athletes in Pretoria, Gauteng, despite generally preserved self-reported shoulder function as measured by the Penn Shoulder Score. These findings indicate that substantial shoulder pain may be present even in the absence of clinically diagnosed injury or marked functional limitation, suggesting that symptoms may reflect early-stage or subclinical loading responses in this population.

The high prevalence of shoulder pain observed in this study is consistent with existing CrossFit literature, which frequently identifies the shoulder as one of the most affected regions. International studies report shoulder injury or pain prevalence ranging between approximately 20% and 40% among CrossFit athletes, with the shoulder consistently ranking as the most vulnerable joint during high-intensity and overhead-based training.^[4,6,10]^ However, most of these studies focus on injuries resulting in time loss from training or medical consultation. In contrast, the present findings suggest that a greater proportion of athletes experience ongoing shoulder pain while continuing to train, indicating that injury-based surveillance models may underestimate the true burden of shoulder-related symptoms in CrossFit populations.

Despite the high prevalence of shoulder pain, participants reported high overall shoulder function. This apparent dissociation between pain and functional performance has been described in other athletic populations and may reflect adaptive mechanisms, pain tolerance, or normalisation of discomfort within high-performance training environments. Silva et al. reported that CrossFit practitioners with shoulder pain maintained functional performance despite altered neuromuscular activation patterns, including reduced lower trapezius activity.^[11]^ Similarly, Summitt et al. found that many CrossFit participants reported shoulder pain while continuing to train, supporting the notion that pain does not necessarily equate to functional limitation in this context.^[10]^

Local South African data further support the vulnerability of the shoulder in CrossFit athletes within the rapidly growing CrossFit community. Studies conducted in KwaZulu-Natal and Pretoria have reported that approximately 38–42% of documented CrossFit-related injuries involve the shoulder.^[12]^ However, these investigations focused on reportable injury events rather than persistent pain or self-reported function. The present study extends this body of knowledge by demonstrating that shoulder pain is highly prevalent even among athletes classified as uninjured, highlighting a substantial burden of symptoms not captured through traditional injury definitions.

Research in other overhead-dominant sports provides additional context. Shoulder pain prevalence among adult swimmers has been reported to range from 19% to 70%, with higher rates observed in younger athletes, likely reflecting cumulative load and early exposure to repetitive overhead activity.^[1–3]^ Similarly, surveillance studies using the Oslo Sports Trauma Research Centre methodology have shown that approximately 22% of elite handball players report persistent shoulder overuse symptoms while continuing to train and compete.^[13]^ These patterns mirror the findings of the present study and reinforce the concept that shoulder pain can exist independently of time-loss injury or overt functional impairment in overhead sports.

The lack of a significant association between training volume and shoulder pain in the present study contrasts with previous research reporting higher injury risk with increased training exposure in CrossFit athletes. [14] This discrepancy may reflect individual load tolerance, neuromuscular adaptation in more experienced athletes, or underreporting of symptoms among those training at higher volumes. It is also possible that cumulative load and movement quality, rather than training volume alone, play a more critical role in the development of shoulder pain. Given the cross-sectional design, causal relationships cannot be established, and longitudinal research is required to further explore these associations.

From a clinical perspective, these findings highlight important limitations of relying solely on time-loss or diagnosis-based definitions of injury. Although all participants were classified as uninjured, the majority reported shoulder pain despite high levels of self-reported function. This underscores the value of incorporating pain- and function-based screening tools into routine monitoring of CrossFit athletes. Early identification of shoulder symptoms may enable targeted interventions to prevent progression to more severe pathology.

### Practical implications

The high prevalence of shoulder pain among uninjured CrossFit athletes underscores the need for routine screening practices that extend beyond injury history alone. The use of validated patient-reported outcome measures, such as the Penn Shoulder Score, alongside clinical assessment, may help clinicians and coaches identify athletes experiencing pain despite preserved function.

Preventive strategies incorporating scapular control, rotator cuff endurance, thoracic mobility, and load management may help address early symptoms and reduce the likelihood of progression to time-loss injury. Encouraging a training culture that supports early reporting of pain may further reduce the burden of shoulder-related conditions in CrossFit populations.

### Limitations

Several limitations should be acknowledged. The use of a convenience sample drawn from CrossFit gyms in Pretoria may limit the generalisability of findings to other regions or competitive levels. The reliance on self-reported pain and training data may introduce recall bias or underreporting, particularly in environments where training through pain is common. Additionally, the absence of objective clinical measures, such as range-of-motion or strength testing, limits the ability to determine whether reported pain corresponds to measurable functional impairment. The cross-sectional design further precludes causal inference regarding the relationship between training characteristics and shoulder outcomes.

### Future directions

Future research should employ prospective longitudinal designs to track the progression of shoulder pain and shoulder function in CrossFit athletes over time. Incorporating objective measures of training load, such as session rating of perceived exertion and wearable monitoring technologies, may provide more precise insight into dose–response relationships. Further investigation is also warranted to determine whether early pain in the presence of preserved function predicts progression to clinically significant shoulder injury.

## Conclusion

This study demonstrates a high prevalence of shoulder pain among uninjured CrossFit athletes in Pretoria, despite generally preserved self-reported shoulder function. These findings highlight the limitations of injury definitions based solely on time loss and emphasise the importance of pain- and function-based screening in high-load training environments. Routine screening and targeted preventive strategies may help mitigate the progression of shoulder symptoms and reduce the long-term burden of shoulder-related conditions in CrossFit athletes.

## Figures and Tables

**Table 1:** Participant demographics and training characteristics (n = 68)

Variable	n (%) or Mean ± SD
Total recruited	70
Valid responses (pain)	68
Age (years)	32.9 ± 9.7
Sex (male/female)	36 (53%) / 32 (47%)
CrossFit experience (years)	4.6 ± 3.1
Training frequency (sessions/week)	5.0 ± 2.6

SD, standard deviation

**Table 2:** Prevalence and severity of shoulder pain (n=68)

Pain category (NPRS)	N	Valid responses
None (0)	16	23%
Low (1–3)	28	41%
Moderate (4–6)	14	21%
High (7–10)	10	15%
Pain present (≥1)	52	76%

NPRS, Numeric Pain Rating Scale

Pain present represents all participants reporting pain (NPRS ≥1) reporting pain (NPRS ≥1).

**Table 3:** Shoulder function outcomes assessed using the Penn Shoulder Score (n = 70)

Penn Shoulder Score domain	Score range	Mean ± SD
Pain subscore	0–30	26.0 ± 4.7
Satisfaction subscore	0–10	7.2 ± 2.7
Function subscore	0–60	54.7 ± 6.9
Total PSS score	0–100	87.8 ± 12.2

PPS, Penn Shoulder Score. Higher scores indicate better shoulder health. Pain sub score (0–30), Satisfaction sub score (0–10), Function sub score (0–60), Total score (0–100)
